# The Online Sale of Antibiotics for Veterinary Use

**DOI:** 10.3390/ani10030503

**Published:** 2020-03-17

**Authors:** Juan F. Garcia, M. Jose Diez, Ana M. Sahagun, Raquel Diez, Matilde Sierra, Juan J. Garcia, M. Nelida Fernandez

**Affiliations:** 1 Department of Mechanical, Informatics and Aerospatiale Engineering, University of Leon, 24071 Leon, Spain; jfgars@unileon.es; 2 Department of Biomedical Sciences, Institute of Biomedicine (IBIOMED), Veterinary Faculty, University of Leon, 24071 Leon, Spain; mjdiel@unileon.es (M.J.D.); rdielz@unileon.es (R.D.); msiev@unileon.es (M.S.); jjgarv@unileon.es (J.J.G.); mnferm@unileon.es (M.N.F.)

**Keywords:** antimicrobials, antibiotics, online sale, prescription, livestock, pets, veterinary, resistance, health care

## Abstract

**Simple Summary:**

Access to antibiotics online endangers its responsible use, increasing the risk of bacterial resistance emergence. The objective of this study was to assess the possibility of purchasing antibiotics for veterinary use on the internet, evaluating the availability of those classified as highest priority critically important antimicrobials (HP-CIA), and if a prescription is required. The Google and Bing search engines and both simple and complex search strings in Spanish and in English were used. The simple search string was “buy veterinary antibiotics”. Complex searches used wildcards and specific syntax. The searches carried out in Spanish revealed that 50% of websites operated in South America and 65% of websites did not require a valid prescription. For the searches in English, 57% of websites operated in the United States of America (USA) and 55% of them did not require a prescription. Our study shows that veterinary antibiotics are easily available for purchase online without a prescription.

**Abstract:**

Antibiotics are essential medicines against infectious diseases in both humans and animals. An inappropriate use of antibiotics can impair animal health and enhance the risk of bacterial resistance, as well as its transfer from animals to humans. The objective of this study was to assess the possibility of purchasing antibiotics for veterinary use on the internet, to evaluate if a prescription is required, and to determine the availability of drugs classified as the highest priority critically important antimicrobials (HP-CIA). The Google and Bing search engines and both simple and complex search strings in Spanish and in English were used. The simple search string was “buy veterinary antibiotics”. Complex searches used wildcards and specific syntax. The searches carried out in Spanish revealed that 50% of websites operated in South America, and 65% of websites did not require a valid prescription. Fluoroquinolones were offered in 84% of these websites (45% without prescription), macrolides were offered in 63% of these websites (43% without prescription), and 3rd– and 4th–generation cephalosporins in 54% of these websites (38% without prescription). For the searches in English, 57% of these websites operated in the United States of America (USA), and 55% of them did not require a prescription. Fluoroquinolones were offered in 79% of these websites (49% without prescription), macrolides were offered in 72% of these websites (45% without prescription), and 3rd– and 4th–generation cephalosporins were offered in 49% of these websites (27% without prescription). Therefore, it is easy to illegally access antibiotics via the internet.

## 1. Introduction

Antibiotics are essential medicines against infectious diseases in both humans and animals. In veterinary medicine, their use is complex, and they have different methods of application, depending on the context and animal species considered. While for companion animals it follows a similar process as a prescription for humans, for food-producing animals the control of bacterial infections can be achieved by means of therapeutic, metaphylactic, and prophylactic treatments after a veterinary prescription. Regarding growth promotion, in the European Union (EU), antibiotic growth promoters were withdrawn in 2006 [1], and in the United States of America (USA), their use has been curtailed since 2017, according to the new rules established by the Food and Drug Administration (FDA) [2].

There is no doubt that antimicrobial use in veterinary medicine has resulted in healthier and, in the case of food-producing species, more productive animals, ensuring the welfare and health for both animals and humans. Over the past decade, increasing attention has been placed to the appropriate use of antimicrobials for both humans and veterinary medicine. The use of antimicrobials, especially misuse or overuse, may promote the selection of resistant bacteria and resistance genes, as well as increasing the risk of new resistant bacteria emerging [3,4].

Resistance has become a global issue and has increased public health awareness due to the risk of nontreatable disease in humans. Although a reliable estimate on the global burden of antimicrobial resistance is difficult to establish, as data are not systematically collected, it has been calculated that, in Europe alone, resistant bacteria in hospital infections may result in approximately extra 25,000 deaths each year, causing €1.5 billion in healthcare costs and productivity losses [5]. It is estimated that 700,000 deaths worldwide may be caused by antimicrobial resistance yearly [6,7].

Antimicrobial resistance should be viewed from a “One Health” perspective [8,9], addressing human and animal health together. Resistant bacteria and resistance genes may be disseminated between animals and humans through various channels, such as animal/human contact, via the food chain, and in the surrounding environment [10,11]. In the case of food-producing animals, although an overall decline in sales (mg/PCU (population correction unit)) of 32.5% was observed in the EU from 2011 to 2017 [12], antimicrobial consumption in several countries is high and increasing. This fact may be due to the growing demand for animal proteins by consumers, epidemiological situations demanding adequate animal treatment, or variations between countries in the occurrence of bacterial diseases, in the composition of the animal population and in production systems [12,13,14]. Global antimicrobial consumption in animal husbandry was estimated at more than 63,000 (±1500) tons in 2010 and is expected to rise by 67% by 2030 [13]. This consumption varied considerably between countries, achieving 318 mg/PCU in China in 2013 [14] and ranging in Europe alone from 3.1 mg/PCU in Norway to 423.1 mg/PCU in Cyprus in 2017 [12].

The World Health Organization (WHO) has defined five groups of antimicrobials as “highest priority critically important antimicrobials” (HP-CIA) (3rd-, 4th-, and 5th-generation cephalosporins, fluoroquinolones, glycopeptides, macrolides, as well as polymyxins) [15]. These substances are considered essential for the treatment of specific infections in humans, and their use in livestock should be restricted. The World Organisation for Animal Health (OIE) considers three of these groups (3rd- and 4th-generation cephalosporins, fluoroquinolones, and macrolides) as “Veterinary Critically Important Antimicrobial Agents” (VCIA) [16]; the Antimicrobial Advice ad hoc Expert Group (AMEG) categorization considers glycopeptides as Category A (“Avoid”) and 3rd- and 4th-generation cephalosporins, fluoroquinolones, and polymyxins as Category B (“Restrict”) in veterinary medicine [17,18].

The WHO global action plan for tackling antimicrobial resistance has defined specific objectives for international antimicrobial stewardship programmes, in order to achieve effective treatments with a minimum of adverse effects [19]. These objectives, which have been undertaken by the FDA, the European Medicines Agency (EMA), and other medicines agencies, include enforcing regulations on the quality, distribution, and use of these drugs, giving priority to over-the-counter sales and antibiotics obtained via the internet [11]. In the EU, human antimicrobial consumption was hardly changed during the period 2009–2018, although significant reductions were observed in some countries [20], whereas in food-producing animals consumption of antimicrobials significantly dropped from 2011 to 2017 [12]. In 2014, the average antimicrobial consumption was higher in food-producing animals (152 mg/kg) than in humans (124 mg/kg), but the opposite trend applies to the median values of consumptions in food-producing animals and humans (67 and 118 mg/kg, respectively). In 18 of 28 European countries, antimicrobial consumption was lower or much lower in food-producing animals than in humans [21].

Health authorities are in charge of establishing regulations on the use of antibiotics under prescription. In the EU, regulations and private guidelines have been the main instrument to limit antibiotic use in animal production. However, it can be very difficult to control their sales online [22]. The use of the internet has rapidly expanded over the last few years, exponentially increasing the possibilities not only to access health information but also to purchase a variety of drugs. Online sales are so large that the FDA has created an entire section on its website for buying medicines over the internet [23]. Many countries have limited the acquisition of antibiotics to prescription only. The EU 6/2019 Regulation allows for online sales of veterinary medicines not subject to prescription within the EU, and the sales of prescription medicines are allowed only to people in the same country [24]. Moreover, it should be noted that acquiring medicines via the internet is an important way of putting one’s health at risk, and in many cases, the websites are illegal, or the medicines are falsified [25].

Overall information on these digital selling platforms or websites is scarce, and no data are available on the sales volume of veterinary medicines. It has been estimated that 96% of global online pharmacies operate illegally by failing to comply with regulatory and safety requirements or professional, legal, and ethical principles [26].

Thus, the purpose of this interdisciplinary study, in which pharmacology and informatics investigators collaborated, is to assess the availability of antibiotics for veterinary use over the internet by evaluating the following aspects: a) if a prescription is required, b) whether the quantities that can be purchased are limited, c) the availability of the drugs classified as the HP-CIA, and d) their shipping countries, among others. This study contributes to a better knowledge of the availability of antibiotics outside of legally controlled prescriptions.

## 2. Materials and Methods

A multidisciplinary working group that included both pharmacology experts (pharmacology professors from the Veterinary Faculty at the University of León, Spain: M.S., J.J.G., M.J.D., A.M.S., R.D., and M.N.F.) and a member with expertise in cybersecurity (the Department of Mechanical, Informatics and Aerospatiale Engineering: J.F.G.) took part in the study. The search of the website pages was programmed and carried out by J.F.G. The pharmacology members of the team reviewed and evaluated by pairs the data included in the captured information. Any potential disagreement was resolved by all members.

The characteristics of the programming developed and the results obtained will be explained below in the corresponding sections, presenting first the technological aspects and then the information found.

### 2.1. Search Engines

To collect all the information, we used the Google and Bing search engines, which together account for 93.5% of the market share of the worldwide search engine market, according to Statcounter [27]. These two search engines, in addition to being two of the most popular search engineers in the world, play a major role in how people address medical needs [28].

### 2.2. Search Terms

For our study, we used both simple and complex search strings. Simple searches are intended to be similar to those of regular users, who want to find a website where they can buy veterinary antibiotics. We used the simple search string “buy veterinary antibiotics”.

Complex searches aim to acquire the greatest amount of information from a search engine, using wildcards and specific syntax. The complex search we used was “%22buy OR purchase%22 %22veterinary OR pet%22 %22antibiotics%22 -%22no antibiotics%22 %22pharmacy OR online store%22”, in which %22 is the URL encoding for a double quote (“), and it is understood by website browsers. The search query contains several blocks, from which a term will be selected later on by the search engine (i.e., Google or Bing), and each of these blocks is delimited by double quotes. Therefore, the search string “%22buy OR purchase%22 %22veterinary OR pet%22” can be read as “buy or purchase” “veterinary or pet”. We used the logical operator (OR) to allow for the search of several alternative terms and the exclusion operator “-” to avoid obtaining results, in which unwanted constructions appeared, such as “no antibiotics”. Thus, we avoided websites that simply sell alternative products to antibiotics.

Searches were carried out in both English and Spanish using the software and tools detailed in the following section. We obtained our results from 4 searches.

### 2.3. Development of the Search

We carried out an automatic extraction of information (through software, see Section 2.4 “Information extraction process” for details) for most of the process. This software not only simplified tasks to be performed but also allowed for greater precision when obtaining results, reducing both fatigue and propensity for human error. Our contribution can thus be summarized as the simple and precise extraction of high volumes of reliable data, which was mainly achieved via software automation.

Thanks in part to the automation achieved, we analyzed more than 3000 websites, a much higher volume than that analyzed in other works, in which researchers analyzed the first few pages of the results of these engines (about 100 or 200 websites in the best case, possibly many fewer if we discard duplicates or advertisements) [29,30].

#### Terms of Service for the Automation of Searches

Contrary to popular belief, the terms of service for search engines do not prohibit automatic searches (using software tools such as bots or crawlers). What they do prohibit is searches that are done using means other than those provided by them (example: the graphical interface of the engine) or those that abuse the system (automatic searches that make excessive use of the service, as thousands of requests per hour could slow or even block services offered to the public) [31].

To perform searches according to these conditions, we developed a specific piece of Java software that performs searches using a web browser (Firefox or Google Chrome), from which it accesses a search engine and directly collects results.

### 2.4. Information Extraction Process

For the extraction of information, the following steps were followed (the first four were carried out automatically by our software, and the last one was completed by the software and one or several human analysts):

1. Access to the search engine. As noted, we used Google or Bing, accessed via the Firefox and Google Chrome browsers, respectively.
2. Search. Search engines group their results by pages, showing a specific and reduced set of results (10 by default) in each of them. To simplify this task and reduce the number of queries that the software must perform, we configured the search engines to print the highest number of search results per page (100 in Google and 50 in Bing).

Search engines usually tell the user that they have found tens or hundreds of thousands or even millions of coincidences (results), but the user is only given a fraction of them (a maximum of between 100 and 200 results). If we configure the search so that each page contains more results, we also increase the number of meaningful results obtained.

In the case of Google, we used an additional plugin that allows all the results to be displayed on the same page, which facilitates their collection. This software makes a maximum of 50 requests per hour to the engine, so as not to saturate it and to comply with the terms of service.

Our system overcomes the influence of the location, where the search was performed. We configured both browsers to work in full private mode, and our software employed specific parameters to prevent search engines from using country-specific website-filtering.

3. Result extraction. According to the terms of service, results (URL) must be collected directly from the engine interface in the browser, which is known as a search engine results page (SERP).

**Figure 1:**
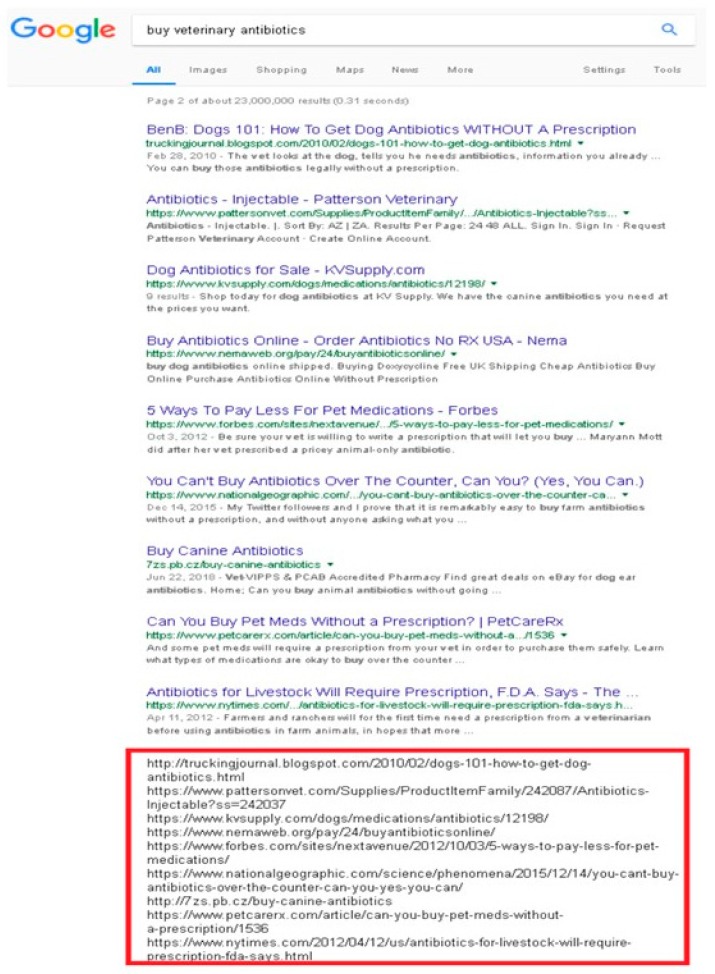
Example of URL extraction at the bottom of a search engine results page (SERP).

Of all the information shown, only the URLs of different websites interested us. In order for our software to obtain these URLs in a simple way, we used specific and complementary software for each scenario: for Google, we equipped the Firefox browser with Greasemonkey (a usescript manager) [32] and an Internet Marketing Ninjas plugin [33] that allowed us to group all the URLs at the end of the SERP (Figure 1).

In the case of Bing, we developed a JavaScript plugin for Google Chrome with functionality analogous to that described for Firefox. At the end of this step, the software extracted a list containing all the websites that search engines provided us with.

4. Websites downloaded for offline analysis. Once we obtained a list of websites that are potential online antibiotics sellers, these sites were analyzed individually to verify that they truly sell antibiotics. To facilitate this task, the free tool Heritrix, a web crawler designed for web archiving by the Internet Archive, was utilized [34].

To speed up this step and to avoid exhausting our resources (especially the available memory in our machines), Heritrix was configured to download only the text content (.txt or .html), discarding the rest of the multimedia content (photos, videos, PDFs, etc.).

5. Analysis of the results obtained. After downloading the websites, all the relevant information they contained was extracted.

This step was the only step that was not fully automatic. While our software is capable of performing basic syntactic analysis (it determines if a certain term, e.g., “amoxicillin”, “veterinary”, or “buy “, appears on a site), the semantic analysis is still performed by pharmacology experts. These researchers evaluated the contexts, in which those terms appear to confirm whether the sites really sell these kinds of substances.

**Figure 2:**
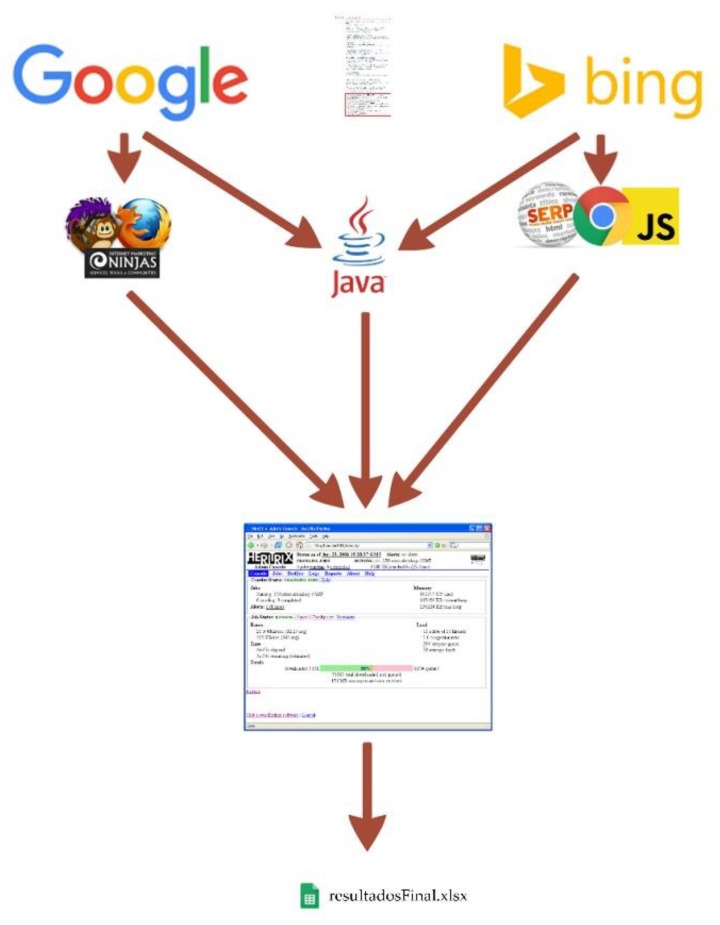
Information extraction process.

All steps were repeated for each search string (simple/complex and English/Spanish) used. Figure 2 summarizes the whole process. The designed software worked for 30 h to obtain results.

### 2.5. Data Obtained from the Website Pages Analyzed

The website pages selected were examined to obtain the following information: a) the country, in which antibiotics were being sold, b) the countries the vendor would sell antibiotics to, c) the expected delivery time to receive antibiotics and how drugs were sent, d) whether antibiotics were available without a prescription, d) the possibility for the customer to purchase HP-CIAs (3rd–, 4th–, and 5th–generation cephalosporins, fluoroquinolones, glycopeptides, macrolides, and polymyxins), e) whether antibiotics were sold for human use, animal use (taking also into account if they were for livestock or pets), and f) the availability of oral, parenteral, and topical dosage forms. This analysis took the authors 5 days to complete.

All information was collected in Microsoft Excel 2016 (Microsoft, Redmon, WA, USA), and a statistical analysis was carried out using SPSS version 24 (IBM Corporation, Armonk, NY, USA). The results obtained were expressed as frequencies and percentages. A chi-square test was performed to compare the number of websites found after carrying out the searches with both search engines and in both languages (Spanish and English). The information obtained from the website pages after the searches using the terms in Spanish and English was also compared by using a chi-squared test. *p* ≤ 0.05 was taken as the level of significance.

## 3. Results

**Figure 3:**
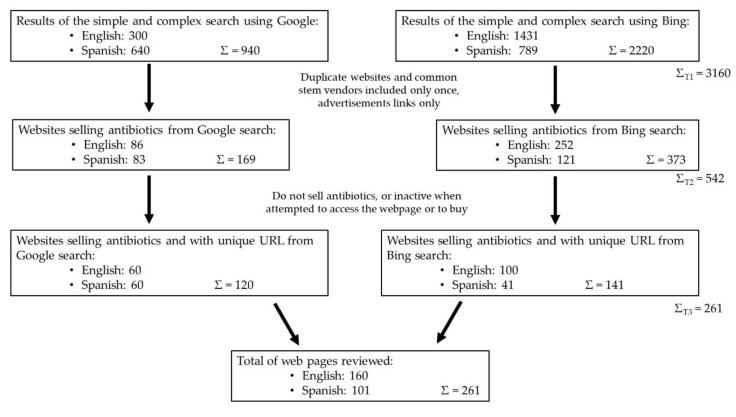
Flow diagram displaying the results from the searches performed in June 2019.

Figure 3 includes the flowchart of the searches performed. In the first phase of the work, 3160 websites were found using the search engines Google and Bing, with search terms in English and in Spanish. The results obtained for both variables were different. The variables *search engine* and *language* were related (*p* < 0.0001). Further, in the second phase, after excluding duplicate websites and those that were advertisement links, and after including common stem vendors only once, 542 websites were selected. The variables *search engine* and *language* were still significantly related (*p* = 0.0002). Finally, in the third phase, after removing all websites that did not sell antibiotics or were inactive when accessed, 261 websites were analyzed in detail, and 160 and 101 of them were obtained after the searches carried out in English and Spanish, respectively, using both search engines. The search results were dependent on the search engine and the language used (*p* = 0.0005).

Regarding the location, most of the vendors identified by the search carried out in Spanish operated in South America (50%), mainly from Brazil (13%), Argentina (11%), and Chile (8%). In Europe (19%), most were from Spain (16%) and France (2%), and the rest were from Mexico (13%).

However, most of the vendors found after the search in English operated in the USA (57%). In Europe (11%), most were from the UK (7%), and none were from Spain. The remainder were from Canada (9%), Australia (4%), and India (4%). No site was from South America, and one was from Mexico.

Table 1 includes the information obtained on the countries the vendor would sell antibiotics to, the expected delivery time to receive antibiotics, the payment method, and how drugs were sent.

In most web pages, sales were restricted to national deliveries (37% and 43% of those found with the searches carried out using Spanish and English terms, respectively). It is noteworthy that in 14% and 13% of the websites (via Spanish and English searches, respectively) information on the countries antibiotics were sold to was not given, mainly due to the obligation of customers to register to obtain such information, a procedure that we did not complete. In the same way, many times, information regarding shipping times or methods or payment methods was not available. Significant differences (chi-squared test) were found for all these aspects between the Spanish and English searches (Table 1).

Information on the need for a prescription to buy antibiotics, whether the quantities of antibiotics sold were limited, who could buy the drugs, and the number of different compounds sold are shown in Table 2.

A prescription was clearly required by 18% and 36% of the vendors using Spanish and English searches, respectively. The largest numbers of websites that did not require a prescription and those asked for it were both found in the USA (51 of 88 vendors and 27 of 58 vendors, respectively).

In many websites (61%), the quantity of drugs was not limited to a certain quantity of packages. This percentage was the same for the websites located using the terms in Spanish or in English. Most vendors sold drugs to private customers: 74% of the website pages obtained after searching in Spanish, and 87% of those located after searching in English sold drugs to private customers. The majority of the websites found after Spanish (66%) or English searches (70%) offered more than 10 different antibiotics.

Table 3 shows the results obtained after determining whether antibiotics were sold for human or animal use (also taking into account if they were for livestock or pets). We found a wide range of patients, for whom these medicinal products were intended.

After reviewing the websites located using Spanish terms, we noted that most websites advertised oral antibiotics (81%) and an important number of them (61%) offered parenteral antibiotics, as well as topical drugs (37%). The English search results produced similar results: 88% of the website pages offered oral antibiotics, 49% of the website pages offered topical ones, and 37% of the website pages offered parenteral drugs.

Finally, we analyzed the HP-CIA that the visited websites were able to supply. After reviewing the sites located with the searches in Spanish, most of them were seen to offer fluoroquinolones, macrolides, and 3rd– and 4th–generation cephalosporins, while the offers were lower for polymyxins and very low for glycopeptides. After the searches with the terms in English, similar results were obtained for fluoroquinolones, macrolides, 3rd– and 4th–generation cephalosporins, polymyxins, and glycopeptides with higher offers. There were no significant differences between the proportions of searches of websites in Spanish and English for each of the antimicrobial groups. Table 4 includes the percentages of websites, where HP-CIA were available without a prescription.

## 4. Discussion

According to the results obtained, there is a possibility to purchase antibiotics from 47% of the websites located with the searches in Spanish and 30% of those chosen from the searches in English for production animals (horses included). All websites from Spain (8 entries) only sold antibiotics to veterinary professionals. This fact is undoubtedly related to Spanish and EU legislations [22,35].

It should be noted that some online sales of veterinary medicines are legal and some medicines can be obtained through legally registered platforms. For example, in the UK, accredited internet retailers can dispense veterinary drugs with a valid prescription, and in other countries, gross sellers can sell them online to veterinary professionals. In this sense, the EU is now designing a common logo to identify websites offering veterinary medicinal products for sale at a distance, and state members will have to list retailers established in a certain country, who are permitted to offer veterinary medicinal products for sale at a distance. However, problems arise, when antibiotics are available to purchase online without a valid prescription, which exposes the population to a variety of potential risks. In our study, we have found that it is easy to access antibiotics online without control.

On the other hand, the European Commission (EC) published guidelines for the prudent use of antimicrobials in veterinary medicine [36]. The purpose of these guidelines is to provide practical guidance for member states on the development and implementation of strategies to promote the prudent use of antimicrobials, especially antibiotics, in veterinary medicine. The reduction of the uncontrolled sale of antimicrobials is a desirable objective to contain antimicrobial resistance [37]. In this sense, the Federation of Veterinarians of Europe already proposed in 2013 that online antimicrobial sales should be banned [38].

Antimicrobial drugs used in animals and humans, although not identical, are very similar [39]. More specifically, antimicrobial-resistant bacteria in animals and humans are closely related [40,41], and antimicrobial resistance genes are identical in many cases [42,43].

The searches carried out automatically by our software, using terms in English and in Spanish, found websites that sold antibiotics for humans, because the websites did not clearly indicate the purpose of drugs; they only published that they sold antibiotics without a prescription. Thus, a significant percentage of antibiotics sold on the websites analyzed were intended for humans (33% in the searches in Spanish and 38% in the searches in English). We also considered these websites among the selected websites, since sometimes human medicines are used to treat animals, mainly pets, and this would be an easy way to obtain antibiotics. On websites dedicated to pets, as well as discussion forums and blogs, it is easy to find information and advice on the use of human medicines, including antibiotics [43].

A significant number of the reviewed websites sold antibiotics for pets. Considering all the aspects evaluated in the search (sale only for dogs and cats, all animal species, and many pets), 62% of the websites in Spanish and 50% of the websites in English sold antibiotics for pets.

Studies on resistance to antibiotics carried out in animals, the transfer of this resistance to humans, and the measures taken to prevent and avoid such resistance are mainly focused on food-producing animals. Nevertheless, antimicrobial resistance is a serious health problem that involves all species and does not recognize any limits between humans, animals, and the environment; in addition, there is close contact and microbial exchange in animal-owning households [44,45].

This aspect is of interest for related professionals, and, in recent years, several articles and the opinions of experts have been published in this regard [39,41,43,46,47,48]. Recently, Currie et al. published an article, in which for the first time experts in veterinary health established an expert consensus at a national level (the UK) about which aspects of veterinarian practice should be targeted to antimicrobial resistance and antimicrobial use in companion animals [43].

Our study has several limitations, including not being able to determine the amount of antibiotics (controlled and not controlled by a veterinarian) obtained from an online purchase. However, we have achieved our purpose of extracting global knowledge of available places online to acquire antibiotics for veterinary use. The design of the search procedure allowed us to identify the sales of antibiotics embedded and hidden deep within websites, of which the scopes, descriptions, or sections were completely unrelated to antibiotics, such as agricultural machinery or seed-selling domains.

The public health authorities, regulators, and regulating agencies of different countries direct their efforts to stop the development of resistance by strictly regulating the sales of antibiotics by using prescriptions and establishing guidelines for the proper use of antibiotics by veterinarians. However, they must also direct their efforts towards controlling the sources of antibiotics obtained without a prescription [42], either for animals or humans.

The WHO and other international organizations specifically recognize the risk posed by illicit online pharmacies, which provide digital platforms for illegal manufacturers in the transnational distributions and sales of antibiotics [48,49,50].

Several studies on this subject have suggested the need for increased health communication, promotion, and education initiatives to better inform the public about the dangers of purchasing antibiotics online, as well as the need to establish sales control procedures [51,52].

The increased regulation of internet sites beyond controlled substances to include antibiotics appears warranted and necessary. The latter will be essential for antibiotics considered the HP-CIA by the WHO. We have found that fluoroquinolones, 3rd–, 4th–, and 5th–generation cephalosporins, macrolides, and polymyxins have important presence in websites selling antibiotics. The WHO and the Organization for Economic Co-operation and Development (OECD) previsions 4 and 5 on the development of resistance to these groups of antibiotics and their consequences for human health justify the urgency of action in this regard.

## 5. Conclusions

The study we have carried out is the first to document the availability of antibiotics on websites for veterinary use; future studies are needed to document the scope of antibiotics purchased on the internet to better understand the direct implications for antibiotic resistance.

In summary, the online availability of veterinary antibiotics is very high in Spanish and English contexts, including HP-CIA. The uncontrolled use of antibiotics in animals endangers their health and also impacts human health and the environment. To contribute to the safe and effective use of veterinary antibiotics, collaborations between regulatory agencies and other official organizations related to human and animal health at the international level are essential to promote the “One Health” approach. Control measures for the sales of antibiotics on the internet should be established, with cybersecurity measures as an essential part of this control, as well as proper enforcement of legislation and an increase in the penalties for illegal trade.

## Figures and Tables

**Table 1:** Characteristics of the web pages reviewed after Spanish (*n* = 101) and English (*n* = 160) searches regarding the countries the vendor would sell antibiotics to, the expected delivery time to receive antibiotics, the payment method, and how they were sent.

Charac-Teristics	Percentage of Websites								*p*-Value ^1^
	Spanish Search				English Search				
No. of countries, where vendors sold antibiotics	Only national deliveries	2–10 countries	>10 countries	N.A.	Only national deliveries	2–10 countries	>10 countries	N.A.	
	37	24	25	14	43	6	36	15	<0.0001
Shipping time	1–2 days	3–7 days	>7 days	N.A.	1–2 days	3–7 days	>7 days	N.A.	
	21	3	12	64	7	12	33	48	<0.0001
Payment mode	Credit card	Several options	N.A.		Credit card	Several options	N.A.		
	33	32	35		48	32	20		0.013
Shipping method	Airmail	Postal services	Other systems	N.A.	Airmail	Postal services	Other systems	N.A.	
	13	10	29	48	3	22	27	48	0.003

N.A.: information not available; ^1^ chi-squared test.

**Table 2:** Characteristics of the website pages reviewed after Spanish (*n* = 101) and English (*n* = 160) searches regarding the need for a prescription to buy antibiotics, whether the quantities sold were limited, who could buy drugs, and the number of different compounds sold.

Charac-Teristics	Percentage of Websites								*p*-Value ^1^
	Spanish Search				English Search				
Prescription required	Yes	No	N.A.		Yes	No	N.A.		
	18	65	17		36	55	9		0.003
Sold drug quantities limited	Yes	No	N.A.		Yes	No	N.A.		
	10	61	29		26	61	13		<0.0001
Who could purchase antibiotics	Only veterinary professionals	Particular customers	N.A.		Only veterinary professionals	Particular customers	N.A.		
	11	74	15		3	87	10		0.013
No. of antibiotics sold	<3	3–10	>10	N.A.	<3	3–10	>10	N.A.	
	7	24	66	3	6	15	70	9	0.111

N.A.: information not available; ^1^ chi-squared test.

**Table 3:** Characteristics of the website pages reviewed after Spanish (*n* = 101) and English (*n* = 160) searches regarding patients, for whom antibiotics are intended.

Type of Patients	Percentage of Websites		*p-*Value ^1^
	Spanish Search	English Search	
All animal species	39	16	0.364
Only humans	22	30	0.157
Humans and animals	11	8	0.436
Only dogs and cats	16	12	0.343
Many pets, including dogs and cats	3	13	0.006
Food producing animals	6	4	0.400
Horses	2	10	0.013
N.A.	1	7	0.028

N.A.: information not available; ^1^ chi-squared test.

**Table 4:** Characteristics of the websites, where highest priority critically important antimicrobials (HP-CIA) were available (via the Spanish (*n* = 101) and English (*n* = 160) searches).

HP-CIA	Percentage of Websites that Were Able to Supply Drugs		*p*-Value ^2^
	Spanish Search	English Search	
Fluoroquinolones	84 (55 ^1^)	79 (49 ^1^)	0.34
Macrolides	63 (43 ^1^)	72 (45 ^1^)	0.15
3rd–and 4th–generation cephalosporins	54 (38 ^1^)	49 (27 ^1^)	0.42
Polymyxins	15 (10 ^1^)	16 (10 ^1^)	0.87
Glycopeptides	5 (2 ^1^)	11 (7 ^1^)	0.08

^1^ without prescription; ^2^ chi-squared test.
